# Physician's Compliance to Clinical Practice Guidelines and Outcomes of Patients With Invasive Candidiasis in a University Hospital in Thailand

**DOI:** 10.1111/myc.70094

**Published:** 2025-07-21

**Authors:** Nantaporn Pirogard, Piriyaporn Chongtrakool, Darunee Lertsudkanung, Methee Chayakulkeeree

**Affiliations:** ^1^ Division of Infectious Diseases and Tropical Medicine, Department of Medicine, Faculty of Medicine Siriraj Hospital Mahidol University Bangkok Thailand; ^2^ Department of Medicine Uttaradit Hospital Uttaradit Thailand; ^3^ Department of Microbiology, Faculty of Medicine Siriraj Hospital Mahidol University Bangkok Thailand; ^4^ Department of Research, Faculty of Medicine Siriraj Hospital Mahidol University Bangkok Thailand

**Keywords:** *Candida*, candidemia, candidosis, quality management, systemic infection

## Abstract

**Introduction:**

Invasive candidiasis is a life‐threatening fungal infection associated with high mortality rates. Adherence to clinical practice guidelines (CPG) has been shown to improve patient outcomes. This study aimed to evaluate physician compliance with CPG following the implementation of care bundles and locally developed CPG and to assess the impact of CPG implementation on patient mortality.

**Methods:**

This quasi‐experimental study utilised a historical cohort control design. Candidemia patients treated at Siriraj Hospital in Bangkok, Thailand, from November 2021 to April 2024 were enrolled. A prospective cohort group received CPG for invasive candidiasis, modified from ESCMID recommendations, covering eight facets. Education care bundles, including clinical policy, training, infographic sheets, leaflets and SMS alerts, were also implemented. Each CPG item was scored as 0, 1 or 2, representing non‐compliance, partial compliance and full compliance, respectively. A total compliance score below eight indicates poor compliance. Physician compliance and 30‐day mortality rates were analysed.

**Results:**

A total of 112 patients were included in the study: 56 in the historical control group and 56 in the prospective intervention group. Both groups exhibited similar baseline characteristics and risk factors for candidemia. Following the implementation of the CPG and care bundles, physician compliance significantly improved across several metrics. Notable increases were observed in: initiating anti‐fungal therapy within 24 h (OR = 6.00, 95% CI [2.41–14.96], *p* < 0.001), receipt of appropriate anti‐fungal therapy, specifically with echinocandins or amphotericin B (OR = 9.17, 95% CI [1.11–75.96], *p* = 0.03), catheter removal or source control within 48 h (OR = 37.17, 95% CI [4.68–295.39], *p* < 0.001), obtaining blood cultures at least every other day (OR = 19.15, 95% CI [7.35–49.86],*p* < 0.001), continuing anti‐fungal therapy for at least 14 days after the first negative culture (OR = 3.30, 95% CI [1.42–7.67], *p* = 0.005), conducting echocardiography (0% vs. 16.1%, *p* = 0.003), performing fundoscopy (OR = 5.24, 95% CI [1.79–15.30], *p* = 0.001). There was a significant improvement in compliance scores, with ≥ 8 being more prevalent in the intervention group compared to controls (OR = 5.39, 95% CI [1.98–14.69], *p* < 0.001). The mean compliance score was 8 ± 2 in the control group and 11 ± 2 in the intervention group (*p* < 0.001). Additionally, the all‐cause 30‐day mortality rate decreased significantly from 55.4% in the control group to 35.7% in the intervention group (OR = 0.45, 95% CI [0.21–0.96], *p* = 0.04).

**Conclusions:**

The implementation of CPG and care bundles for invasive candidiasis significantly enhanced physician compliance and improved patient survival. These findings support the continued adoption of CPG and care bundles in the management of invasive candidiasis.

## 1 Introduction

Invasive candidiasis is a serious and highly morbid fungal infection with a significant global impact. Published data indicate an overall mortality rate ranging from 44% to 71%, even in resource‐rich countries [1, 2, 3, 4, 5]. Improving the management of these life‐threatening fungal infections is crucial for enhancing the capacity of healthcare systems. A recent study in Thailand revealed a notably high mortality rate of approximately 71% among patients with candidemia [5]. This underscores the urgent need for effective management strategies to improve patient outcomes in candidemia.

Diagnosis and treatment of candidemia were guided by the current recommendations from the European Society of Clinical Microbiology and Infectious Diseases (ESCMID) [1, 6] and the Infectious Diseases Society of America (IDSA) guidelines [7]. Despite these established guidelines, physician compliance has been inconsistent, and mortality rates associated with candidemia have not decreased over the past decade [8].

Recent studies have shown a strong association between higher physician adherence to current guidelines for candidemia and lower mortality rates. One study found that adherence to ESCMID guidelines reduced the 30‐day mortality rate among patients with candidemia from 36% to 9% [9]. Additional trials have demonstrated that adherence to management bundles for candidemia significantly enhances survival benefits [10]. Key factors for improving patient outcomes include prompt diagnosis, early initiation of appropriate anti‐fungal therapy and timely source control [3, 10, 11].

The aim of this study was to evaluate physician compliance with the locally modified clinical practice guidelines (CPG) and care bundles implemented for invasive candidiasis as well as to assess the overall mortality in patients with candidemia. The CPG were adapted from ESCMID recommendations, aligning with our specific clinical scenarios and available resources.

## 2 Materials and Methods

### 2.1 Study Design

This quasi‐experimental study utilised a historical cohort control design and enrolled candidemia patients admitted to Siriraj Hospital in Bangkok, Thailand. The historical control group comprised patients hospitalised from November 2021 to February 2023, prior to the implementation of the CPG. The prospective cohort included patients admitted between March 2023 and April 2024, after the CPG was implemented. The study received approval from the Siriraj Institutional Review Board (SiRB; COA number *Si* 847/2022) and informed consent was obtained from all patients in the prospective intervention group. This study was registered with the Thai Clinical Trials Registry (ID number: TCTR20221116001).

### 2.2 Study Population

All candidemia patients aged 18 years or older with a positive blood culture for *Candida* spp. were enrolled in the study. Patients were excluded if they had recurrent candidemia, if their medical records were incomplete, or if they had died or were expected to die within 24 h after the diagnosis of invasive candidiasis.

### 2.3 Data Collection

Patient demographic and clinical data collected included age, gender and medical comorbidities such as diabetes mellitus, cardiac disease, chronic kidney disease, chronic lung disease, chronic liver disease, HIV, haematological malignancy, solid malignancy, transplantation and autoimmune disease. Risk factors for candidemia were also documented, including neutropenia (absolute neutrophil counts < 500 cells/mm^3^), septic shock, intensive care unit (ICU) admission, mechanical ventilation, central venous catheter (CVC) insertion, parenteral nutrition, haemodialysis, peritoneal dialysis, recent abdominal surgery within the past month, urinary catheter, prosthesis, corticosteroid use, presence of yeast in non‐sterile body samples, broad spectrum antibiotics within the past month and azole exposure within the past month.

The site of infection was classified as either primary fungemia or secondary bacteremia resulting from deep‐seated candidiasis, such as intra‐abdominal infections. All *Candida* species isolated from blood cultures were identified, and anti‐fungal susceptibility testing was performed. However, our fungal laboratory does not routinely perform anti‐fungal susceptibility testing for *Candida albicans* , as it is assumed that all strains are susceptible to fluconazole, based on our hospital anti‐biogram.

The locally developed Clinical Practice Guideline (CPG) consisted of eight key items. These included:

1. Infectious disease (ID) consultation
2. Initiating anti‐fungal therapy within 24 h
3. Appropriate initial anti‐fungal therapy with an echinocandin or amphotericin B products
4. Catheter removal or source control within 48 h
5. Obtaining blood cultures at least once every other day
6. Duration of treatment of at least 14 days after the first negative blood culture
7. Echocardiogram for persistent positive blood cultures beyond 5 days
8. Ophthalmological examination within 2 weeks of diagnosis

For the historical control group, data were collected through a review of electronic medical records to obtain baseline characteristics and CPG‐related items. In contrast, data for the intervention group were collected prospectively following the implementation of the CPG and care bundles, with informed consent obtained from all participants.

Additionally, we developed a scoring system to evaluate physician compliance with the CPG (see Tables S1 and S2). Each CPG item was assigned a value ranging from 0 to 2, allowing for the calculation of compliance scores. A score of 0 indicates non‐compliance, 1 indicates partial compliance, and 2 indicates full compliance with the CPG item. A complete compliance score of 14 indicated adherence to all recommendations, whereas acceptable compliance scores ranged from 8 to 13. Scores below 8 were classified as poor compliance. Treatment outcomes assessed included all‐cause 30‐day mortality, definitive anti‐fungal treatment, duration of anti‐fungal therapy, length of hospital stay and time to resolution of candidemia.

Species identification of *Candida* was conducted based on biochemical characteristics using chromogenic medium (Brilliance *Candida* agar) and the RapID Yeast Plus Panel (Thermo Fisher Scientific, Kent, UK). Some *Candida* isolates required identification via Matrix‐Assisted Laser Desorption/Ionisation Time‐of‐Flight Mass Spectrometry (MALDI‐TOF MS). Anti‐fungal susceptibility testing was performed using the microbroth dilution method with the Thermo Scientific Sensititre Yeast‐One YO9 AST Plate (Thermo Fisher Scientific, Waltham, MA, USA). Minimum inhibitory concentration (MIC) breakpoints were interpreted according to the Clinical Laboratory Standards Institute (CLSI) guidelines.

### 2.4 Outcome and Definitions

Invasive candidiasis is defined as candidemia (positive blood culture for *Candida* spp.) with or without deep‐seated candidiasis (positive *Candida* spp. culture from sterile clinical specimens).

The primary outcome of this study was to evaluate physician compliance with the CPG for invasive candidiasis following the implementation of the care bundle intervention. The secondary outcome was to assess all‐cause 30‐day mortality following the diagnosis of candidemia.

### 2.5 Intervention

**FIGURE 1:**
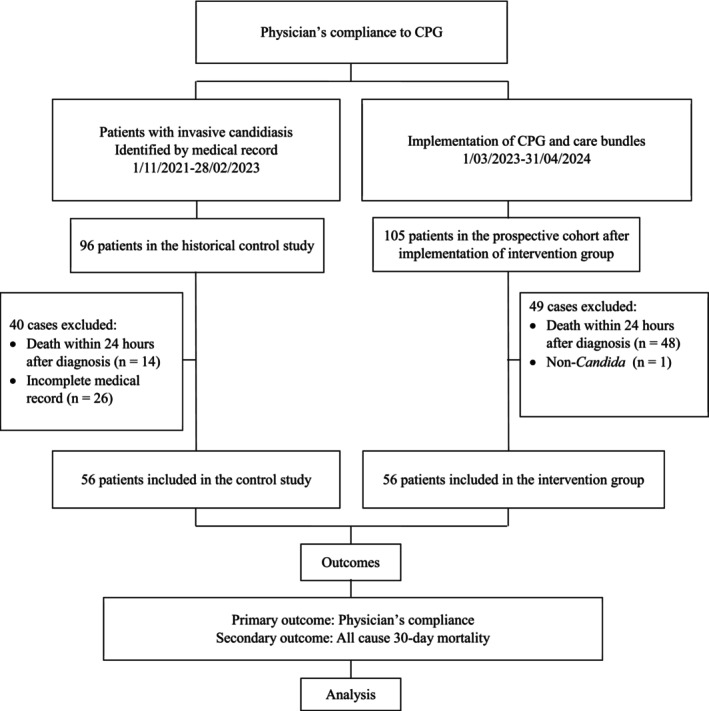
Consort flow diagram of the study.

We implemented CPG for invasive candidiasis and care bundles—including an education programme comprising clinical policy, training, infographic sheets, leaflets and SMS alerts—at Siriraj Hospital 1 month prior to the start of the prospective cohort, as illustrated in Figure 1.

### 2.6 Statistical Analysis

For sample size calculation, we referenced a previous study implementing ESCMID guidelines and estimated that physician compliance would improve by approximately 50%, increasing from 27% to 54% among patients with candidemia [9]. For the secondary outcome, we anticipated that the mortality rate among candidemia patients would decrease from 71% in our previous study to 35% following the implementation of the intervention [5]. With 80% power and an estimated dropout rate of 10%, a two‐sided *p*‐value of less than 0.05 was used to determine the required sample size, which amounted to 112 patients across both groups. Therefore, the target enrolment was set at 56 patients per group (1:1 ratio).

Continuous data are presented as mean ± standard deviation (SD) for normally distributed variables and as median and interquartile range (IQR) for non‐normally distributed variables. Categorical data are reported as frequency and percentage. The Chi‐square test or Fisher's exact test was utilised for comparisons of categorical variables between the two groups. The independent *t*‐test was employed to compare normally distributed continuous data, whereas the Mann–Whitney *U* test was used for non‐normally distributed continuous data. All statistical analyses were conducted using SPSS Statistics (v18.0) software (SPSS Inc., Chicago, IL, USA), and a two‐sided *p*‐value of less than 0.05 was considered statistically significant.

## 3 Results

### 3.1 Patient Characteristics

A total of 112 patients were enrolled in the study (Figure 1). Baseline characteristics and risk factors are detailed in Table 1. The mean age of the participants was 62 years, with 56% being male. The most common underlying conditions included diabetes mellitus (33.9%), cardiac disease (28.6%), solid malignancies (20.5%) and haematological malignancies (18.8%). The risk factors associated with invasive candidiasis included the following: receipt of broad‐spectrum antibiotics (94.6%), retained urinary catheters (74.1%), central venous catheters (65.2%), presence of yeast in non‐sterile sites (64.3%), use of mechanical ventilation (59.8%), ICU admission (57.1%) and septic shock (54.5%). Recent abdominal surgery was noted in 28.6% of patients, while 18.8% were neutropenic. Both control and intervention groups exhibited similar baseline characteristics and risk factors for candidemia, with no statistically significant differences observed.

**TABLE 1:** Characteristics and risk factors of patients with invasive candidiasis.

Characteristics/risk factors	Control group (*n* = 56)	Intervention group (*n* = 56)	*p*
Male gender	32 (57.1%)	31 (55.4%)	0.85
Age (years), mean ± SD	61 ± 21	64 ± 17	0.33
Diabetes mellitus	21 (37.5%)	17 (30.4%)	0.43
Cardiac disease	18 (32.1%)	14 (25.0%)	0.40
Chronic kidney disease	9 (16.1%)	6 (10.7%)	0.41
Chronic lung disease	5 (8.9%)	2 (3.6%)	0.44
Chronic liver disease	2 (3.6%)	2 (3.6%)	1.00
HIV disease	3 (5.4%)	1 (1.8%)	0.62
Haematological malignancy	9 (16.1%)	12 (21.4%)	0.47
Solid malignancy	11 (19.6%)	12 (21.4%)	0.82
Transplantation	8 (14.3%)	5 (8.9%)	0.38
Autoimmune disease	5 (8.9%)	4 (7.1%)	1.00
Others co‐morbidities	34 (60.7%)	39 (69.6%)	0.32
Neutropenia	9 (16.1%)	12 (21.4%)	0.47
Septic shock	34 (60.7%)	27 (48.2%)	0.18
ICU admission	35 (62.5%)	29 (51.8%)	0.25
Mechanical ventilation	38 (67.9%)	29 (51.8%)	0.08
Central venous catheter	38 (67.9%)	35 (62.5%)	0.55
Parenteral nutrition	15 (26.8%)	9 (16.1%)	0.17
Haemodialysis	22 (39.3%)	19 (33.9%)	0.56
Peritoneal dialysis	1 (1.8%)	0 (0%)	1.00
Recent abdominal surgery	17 (30.4%)	15 (26.8%)	0.68
Urinary catheter	46 (82.1%)	37 (66.1%)	0.05
Prosthesis	2 (3.6%)	3 (5.4%)	1.00
Corticosteroid use	10 (17.9%)	8 (14.3%)	0.61
Presence of yeast in non‐sterile body samples	34 (60.7%)	38 (67.9%)	0.43
Receipt of broad‐spectrum antibiotics during the past 1 month	54 (96.4%)	52 (92.9%)	0.68
Azole exposure during the past 1 month	9 (16.1%)	6 (10.7%)	0.41

Abbreviation: ICU, intensive care unit.

### 3.2 Microbiology

Diagnosis and microbiology results are summarised in Table 2. The *Candida* species isolated from the patient's blood samples included *Candida tropicalis* (43.8%), *C. albicans* (34.8%), *Candida parapsilosis* complex (12.5%) and *Candida glabrata* complex (11.6%). The prevalence of *Candida* species did not differ significantly between the groups, except for the *C. glabrata* complex, which was higher in the intervention group compared to the control group (17.9% vs. 5.4%, *p* = 0.04). Anti‐fungal susceptibility results were available for 68 of the *Candida* isolates (60.7%), primarily among non‐*albicans* species. In the intervention group, 38 out of 56 patients (67.9%) had susceptibility results, compared to 30 out of 56 patients (53.6%) in the control group (*p*‐value = 0.12). All isolates were susceptible to echinocandins, and 98.5% were susceptible to amphotericin B. In contrast, only 51.5% and 48.5% of isolates were susceptible to fluconazole and voriconazole, respectively (Table S3).

**TABLE 2:** Diagnosis and microbiology data.

	Control (*n* = 56)	Intervention (*n* = 56)	*p*
Site of infection			
Primary fungemia	46 (82.1%)	48 (85.7%)	0.61
Intra‐abdominal infection with fungemia	10 (17.9%)	8 (14.3%)	0.61
Mixed pathogens^a^	10 (17.9%)	10 (17.9%)	1.00
*Candida* species isolated			
*Candida tropicalis*	24 (42.9%)	25 (44.6%)	0.85
*C. albicans*	20 (35.7%)	19 (33.9%)	0.84
*C. glabrata* complex	3 (5.4%)	10 (17.9%)	0.04
*C. parapsilosis* complex	10 (17.9%)	4 (7.1%)	0.09
Others species	1 (1.8%)	0 (0%)	NA
Perform anti‐fungal susceptibility^b^	30 (53.6%)	38 (67.9%)	0.12

^a^ Mixed pathogens included: Dual candidemia found 20% in the control group ( *C. tropicalis* and *C. albicans* , *C. glabrata* complex and *C. parapsilosis* complex) and 20% in the intervention group ( *C. tropicalis* and *C. albicans* , *C. glabrata* complex and *C. albicans* ) Multidrug‐resistant gram negative pathogens: 40% in control group (20% carbapenem‐resistant *Klebsiella pneumoniae* , 10% *Escherichia coli* , 10% carbapenem‐resistant *Acinetobacter baumannii* ), 60% in the intervention group (30% carbapenem‐resistant *Acinetobacter baumannii* , 10% carbapenem‐resistant *Klebsiella pneumoniae* , 10% *Stenotrophomonas maltophilia* , 10% non‐fermentative gram‐negative bacteria). Gram positive pathogens: 40% in the control group (30% Coagulase‐negative *Staphylococcus*, 10% *Enterococcus. faecium*), 20% in the intervention group (10% *Enterococcus faecium* , 10% methicillin‐susceptible *Staphylococcus aureus* ).

^b^ Non‐*albicans Candida* 32/37 isolates (86.5%) in the intervention group and 25/36 isolates (69.4%) in the control group.

Most patients had primary fungemia, accounting for 83.9% of cases. Mixed pathogens were identified in blood cultures in 17.9% of patients, with the majority of co‐infections being multi‐drug resistant gram‐negative bacteria. Among patients with fungemia, 40% (*n* = 4/10) in the control group and 25.0% (*n* = 2/8) in the intervention group also had positive cultures from sterile intra‐abdominal specimens.

### 3.3 Physician's Compliance Outcomes

Significant improvements in physician compliance with all eight recommendations were observed among the 56 patients in the interventional group (*p* < 0.05). Notably, 100% adherence to infectious disease (ID) consultation was maintained in both groups (Table 3). Beyond ID consultation, significant increases in compliance were noted in all other seven facets of CPG recommendations, including initiating anti‐fungal therapy within 24 h, receipt of appropriate treatment with echinocandins or amphotericin B products, catheter removal or source control within 48 h, obtaining blood cultures at least every other day, continuing anti‐fungal treatment for at least 14 days, conducting echocardiography and performing fundoscopy. Of the nine patients (8.0%) who underwent echocardiography due to persistent positive blood cultures for more than 5 days, only one was diagnosed with endocarditis. Similarly, of the 24 patients (21.4%) who received ophthalmological examinations, only one was diagnosed with endogenous candida endophthalmitis.

**TABLE 3:** Physician's compliance to the clinical practice guidelines (CPG).

Suggestion	Control group (*n* = 56)	Intervention group (*n* = 56)	*p*	Odds ratio (95% CI)
ID consultation	56 (100%)	56 (100%)	NA	NA
Initiation of anti‐fungal therapy within 24 h of diagnosis	28 (50.0%)	48 (85.7%)	0.03	6.00 (2.41–14.96)
Appropriate initial anti‐fungal therapy^a^	48 (85.7%)	55 (98.2%)	< 0.001	9.17 (1.11–75.96)
CVC removal/source control within 48 h	23 (54.8%)	45 (97.8%)	< 0.001	37.17 (4.68–295.39)
Take blood culture every other day	12 (21.4%)	47 (83.9%)	0.005	19.15 (7.35–49.86)
Treatment at least 14 days	31 (55.4%)	45 (80.4%)	0.003	3.30 (1.42–7.67)
Echocardiogram	0 (0%)	9 (16.1%)	0.001	NA
Ophthalmological examination	5 (8.9%)	19 (33.9%)	< 0.001	5.24 (1.79–15.30)
Score, mean ± SD	8 ± 2	11 ± 2	< 0.001	2.16 (1.64–2.85)
Score 13–14	0 (0%)	5 (8.9%)	< 0.001	NA
Score 8–12	34 (60.7%)	45 (80.4%)	< 0.001	NA
Score < 8	22 (39.3%)	6 (10.7%)	< 0.001	NA
Stratified scoring system				
Score ≥ 8	34 (60.7%)	50 (89.3%)	< 0.001	5.39 (1.98–14.69)
Score < 8	22 (39.3%)	6 (10.7%)	< 0.001	Reference

Abbreviations: CVC, central venous catheter; ID, infectious disease; NA, not available.

^a^ Appropriate initial anti‐fungal therapy included receipt of micafungin or amphotericin B products.

Compliance scores were calculated for each patient, with higher scores indicating greater adherence to guidelines. A score of less than 8 was considered indicative of poor compliance. The mean compliance score was 8 ± 2 in the control group and 11 ± 2 in the intervention group (*p*‐value < 0.001). Stringent compliance, represented by scores of 13–14, was observed in 8.9% of patients in the intervention group. There was a significant improvement in physician compliance, with scores of ≥ 8, in the intervention group compared to the control group (OR = 5.39, 95% CI [1.98–14.69], *p* < 0.001).

### 3.4 All Cause 30‐Day Mortality and Other Outcomes

After the implementation of CPG, all‐cause 30‐day mortality was reduced to 35.7% in the intervention group, compared to 55.4% in the control group (*p* value = 0.04), as shown in Table 4. Following fungal identification and anti‐fungal susceptibility testing, micafungin was the most commonly used anti‐fungal treatment in the intervention group (46.4%), followed by fluconazole (35.7%). The intervention group demonstrated a median time to resolution of 2 days, compared to 5 days in the control group. In both groups, treatment was administered for a total of 14 days following the confirmation of negative blood cultures.

**TABLE 4:** Treatment outcomes of patients with invasive candidiasis.

Outcomes	Control group (*n* = 56)	Intervention group (*n* = 56)	*p*
Anti‐fungal treatment			
Micafungin	19 (33.9%)	26 (46.4%)	0.52
Amphotericin B products^a^	9 (16.1%)	7 (12.5%)	0.52
Fluconazole	27 (48.2%)	19 (35.8%)	0.52
Length of stay (days), median (IQR)	38 (21–54)	37 (21–64)	0.48
Resolution of candidemia, *n* (%)	45 (80.4%)	49 (87.5%)	0.30
Time to resolution of candidemia (days), median (IQR)	5.0 (3–7)	2 (2–4)	< 0.001
All cause 30‐day mortality	31 (55.4%)	20 (35.7%)	0.04

Abbreviation: IQR, interquartile range.

^a^ Amphotericin B products include liposomal amphotericin B (1.8% vs. 1.8% in the control and intervention group, respectively) and amphotericin B deoxycholate (14.3% vs. 10.7% in the control and intervention group, respectively).

## 4 Discussion

After implementing clinical practice guidelines (CPG) and care bundles for candidemia and invasive candidiasis, there was a significant improvement in physician compliance across nearly all items, except for ID consultation, where full adherence was observed in both groups. The implementation of CPG and care bundles resulted in a significantly lower rate of all‐cause 30‐day mortality in the intervention group. Previous studies have demonstrated the efficacy of “checklist” intervention bundles on the clinical outcomes of patients with candidemia [10]. Accordingly, guidelines recommend echinocandins as the first‐line treatment for patients with invasive candidiasis due to their broader spectrum of activity, higher fungicidal efficacy against most *Candida* species, and better safety profile [1, 3, 6].

Adherence to international guidelines may be challenging in routine clinical practice, often resulting in suboptimal compliance. The EQUAL Candida score, which incorporates key international recommendations, was developed as a practical tool to assess adherence to these guidelines [12]. However, in our setting, full compliance with international guidelines was not achievable due to limitations in the accessibility and availability of anti‐fungal agents in Thailand. Therefore, local guidelines were adapted from international recommendations to accommodate the specific constraints of our healthcare context. For lifesaving purposes, we recommend using an echinocandin or an amphotericin B product—either liposomal amphotericin B or amphotericin B deoxycholate—as acceptable initial anti‐fungal agents. Despite a substantial number of cases treated with amphotericin B deoxycholate, we observed significantly lower mortality rates in the intervention group. The highest physician compliance scores of 13–14 correlated with a favourable outcome, achieving 100% survival in those cases. Additionally, implementing a short message alert system to notify physicians of positive cultures facilitated early diagnosis of candidemia, leading to prompt management in accordance with the CPG. This proactive, systematic strategy likely played a crucial role in improving patient outcomes and enhanced compliance has the potential to further reduce mortality [9, 10].

Following the implementation of clinical practice guidelines (CPG) with care bundles, all‐cause 30‐day mortality significantly declined by approximately 55% compared to the control group. This outcome aligns closely with results from previous studies [10]. Local epidemiological data on candidemia was essential for developing evidence‐based treatment strategies [11]. Over the past 6 years, studies have indicated that *C. tropicalis* , often exhibiting fluconazole resistance, is the most prevalent species in Thailand [5]. Consequently, fluconazole is not considered an appropriate initial anti‐fungal therapy. In our CPG, we recommended the use of echinocandins or amphotericin B products.

The ESCMID recommends performing echocardiography as soon as possible for all patients with candidemia [1]. In our study, only 16.1% (*n* = 9) of patients underwent echocardiography due to persistent candidemia, with only one patient (1.79%) diagnosed with endocarditis. Although candida endocarditis is a serious complication that necessitates specific and intensive therapy, it occurs in only 2.5%–5.5% of patients with candidemia [9, 13, 14]. Therefore, routine echocardiography is not recommended in our setting. By the time we completed this study, an updated global guideline for the diagnosis and management of candidiasis was published by the European Confederation of Medical Mycology (ECMM) [15]. This latest guideline recommends the use of care bundles in the management of invasive candidiasis, consistent with the approach proposed in our study. These recommendations further support the importance of implementing care bundles in the management of invasive candidiasis.

In our study, only 21.4% (*n* = 24) of participants underwent ophthalmological examinations, and among those, only two patients (8.3%) were diagnosed with endogenous candida endophthalmitis. The IDSA and ESCMID recommend fundoscopic examinations for all cases of candidemia to assess for ophthalmological involvement. Intraocular candidiasis rates can vary widely, ranging from 1.3% to 25% [9, 16, 17]. However, due to the limited benefit of routine fundoscopy, particularly in asymptomatic patients, its overall utility remains unclear.

### 4.1 Limitations

The limitations of our study primarily stem from the lack of a randomised study design, and because it was conducted at a single centre, the external validity of our findings needs further verification. Despite these limitations, our study represents the first evaluation in Thailand of physician adherence to CPG and the outcomes of patients with invasive candidiasis at a tertiary medical centre. To enhance the management of invasive candidiasis, these CPG were adapted from ESCMID guidelines, ensuring they are locally relevant, easy to implement, and effective. Notably, physician compliance with each recommendation was higher than in previous studies [10]. Most importantly, the implementation of these CPG led to improved patient survival.

## 5 Conclusions

The implementation of care bundles and CPG for invasive candidiasis in our study significantly enhanced physician compliance and improved patient survival rates. These findings underscore the importance of adapting and applying systematic care approaches tailored to local settings. Our results support the continued integration of these guidelines into clinical practice to optimise outcomes for patients with invasive candidiasis.

## Author Contributions

**Nantaporn Pirogard:** conceptualization, investigation, visualization, writing – original draft, validation, data curation, methodology, formal analysis. **Piriyaporn Chongtrakool:** visualization, resources, data curation, investigation. **Darunee Lertsudkanung:** investigation, data curation, project administration. **Methee Chayakulkeeree:** conceptualization, funding acquisition, investigation, writing – original draft, writing – review and editing, validation, methodology, supervision.

## Consent

All authors agreed to the publication of the current version of the article.

## Conflicts of Interest

M.C. has received research grants from, is an advisor to, is supported for attending meetings and/or travel or received lecture honoraria from AstraZeneca, Basilea, F2G, Gilead, Janssen, Pulmocide, Merck/MSD, Pfizer, Shionogi and Takeda. All remaining authors have declared no conflicts of interest.

## Supporting information


**Table S1.** Clinical practice guidelines (CPG) for invasive candidiasis.

**Table S2.** Scoring system to evaluate the physician’s compliance to the CPG.

**Table S3.** Anti‐fungal susceptibility prolife of 68 isolates from control and intervention groups.

## Data Availability

The data that support the findings of this study are available on request from the corresponding author. The data are not publicly available due to privacy or ethical restrictions.
