# Supplementary material for: Physician's Compliance to Clinical Practice Guidelines and Outcomes of Patients With Invasive Candidiasis in a University Hospital in Thailand
Source: Mycoses. 2025 Jul 21;68(7):e70094. doi: 10.1111/myc.70094 (PMC12278342; doi:10.1111/myc.70094)
Supplement: Supplementary file 1 — Table S1. Clinical practice guidelines (CPG) for invasive candidiasis. [file MYC-68-e70094-s003.docx]

**Table S1.** Clinical practice guidelines (CPG) for invasive candidiasis

| Recommendations | | | | | | |
| --- | --- | --- | --- | --- | --- | --- |
| 1. | Infectious disease (ID) consultation | | | | **□** Yes | **□** No |
| 2. | Initiation of antifungal therapy within 24 hours of diagnosis | | | | **□** Yes | **□** No |
| 3. | Type of antifungal therapy within 24 hours: echinocandins, liposomal amphotericin B or alternative drug: amphotericin B deoxycholate | | | | **□** Yes | **□** No |
| Candidemia | | | | Intra-abdominal candidiasis | | |
| 4. | Removal of catheter or source control within 48 hours | **□** Yes | **□** No | Source controlled within 48 hours | **□** Yes | **□** No |
| 5. | Obtaining a blood culture at least once every other day | **□** Yes | **□** No |  |  |  |
| 6. | Duration of treatment at least 14 days from the first negative blood culture | **□** Yes | **□** No |  |  |  |
| 7. | Consider echocardiogram if  -Persistent positive blood culture on day 5 after adequate treatment  - Clinical suspected IE  - History of IE, prosthetic valve, IVDU | **□** Yes | **□** No |  |  |  |
| 8. | Consider ophthalmologic exam within 2 weeks after diagnosis | **□** Yes | **□** No |  |  |  |
