# Supplementary material for: Physician's Compliance to Clinical Practice Guidelines and Outcomes of Patients With Invasive Candidiasis in a University Hospital in Thailand
Source: Mycoses. 2025 Jul 21;68(7):e70094. doi: 10.1111/myc.70094 (PMC12278342; doi:10.1111/myc.70094)
Supplement: Supplementary file 2 — Table S2. Scoring system to evaluate the physician’s compliance to the CPG. [file MYC-68-e70094-s002.docx]

**Table S2.** Scoring system to evaluate the physician’s compliance to the CPG

| **Recommendations** | | **Scores** | | |
| --- | --- | --- | --- | --- |
|  |  | **0**  **(non-compliance)** | **1**  **(partial compliance)** | **2**  **(full compliance)** |
| 1. | Infectious disease (ID) consultation | Not done |  | Done |
| 2. | Initiation of antifungal therapy | Start > 48 hr | Start at 24-48 hr | Start < 24 hr |
| 3. | Removal of catheter or source controlled | Perform > 48 hr | Perform at 24-48 hr | Perform < 24 hr |
| 4. | Appropriate initial antifungal therapy | Others | Amphotericin B | Micafungin |
| 5. | Obtaining a blood culture at least once every other day | Not done | Less often | Yes |
| 6. | Duration of treatment at least 14 days from the first negative blood culture | Not done | Less than 14 days | Yes |
| 7. | Consider echocardiogram if persistent positive blood culture on day 5 | Not done | Yes |  |
| 8. | Consider ophthalmologic exam within 2 weeks after diagnosis | Not done | Yes |  |
| **Total score** | | **14** | | |
