# Supplementary material for: Physician's Compliance to Clinical Practice Guidelines and Outcomes of Patients With Invasive Candidiasis in a University Hospital in Thailand
Source: Mycoses. 2025 Jul 21;68(7):e70094. doi: 10.1111/myc.70094 (PMC12278342; doi:10.1111/myc.70094)
Supplement: Supplementary file 3 — Table S3. Anti‐fungal susceptibility prolife of 68 isolates from control and intervention groups. [file MYC-68-e70094-s001.docx]

**Table S3** Antifungal susceptibility prolife of 68 isolates from control and intervention groups

| **Antifungal agents** | **Control group**  **(N=30)** | **Intervention group**  **(N=38)** | **Total**  **(N=68)** |
| --- | --- | --- | --- |
| Amphotericin B | 29 (96.7%) | 38 (100%) | 67 (98.5%) |
| Anidulafungin | 30 (100%) | 38 (100%) | 68 (100%) |
| Caspofungin | 30 (100%) | 36 (100%) | 66 (97.1%) |
| Micafungin | 30 (100%) | 38 (100%) | 68 (100%) |
| Fluconazole | 18 (60.0%) | 17 (44.7%) | 35 (51.5%) |
| Voriconazole | 16 (53.3%) | 17 (44.7%) | 33 (48.5%) |

Minimal inhibitory concentration (MIC) breakpoints were determined according to the Clinical and Laboratory Standards Institute (CLSI) guidelines. For amphotericin B, an MIC ≤ 1 mg/L was considered susceptible.
